# Comparison of pyridostigmine and bisacodyl in the treatment of refractory chronic constipation

**Published:** 2016

**Authors:** Iman Soufi-Afshar, Aliakbar Moghadamnia, Ali Bijani, Sohrab Kazemi, Javad Shokri-Shirvani

**Affiliations:** 1 Department of Internal Medicine, Babol University of Medical Sciences, Babol, Iran.; 2 Department of Pharmacology, Babol University of Medical Sciences, Babol Iran.; 3 Social Determinant of Health Research Center - Health Research Institute - Babol University of Medical Sciences, Babol, Iran.; 4 Cellular & Molecular Biology Research Center, Babol University of Medical Sciences, Babol, Iran.

**Keywords:** Constipation, Chronic constipation, Anti-cholinesterase, Pyridostigmine

## Abstract

**Background:**

Treatment of chronic constipation is creating one of the major problems for doctors and patients. Pyridostigmine increases the gastrointestinal motility through the effects on cholinesterase. It seems that this mechanism can reduce chronic constipation. The aim of this study was to compare the effects of pyridostigmine and bisacodyl on chronic constipation.

**Methods:**

This study was conducted on 68 patients who suffered from chronic constipation. Patients were randomly divided into two groups of Pyridostigmine and bisacodyl in which each consisted of 34 patients, respectively. Bristol stool form score, straining defecation, the time of defecation, the number of defecation per week, sense of incomplete evacuation and self-digitation were collected by means of questionnaires and the data were compared.

**Results:**

Sixty-eight patients with the mean age of 68.12±84.49 were studied. The mean difference in the frequency of defecation per week, VAS score, the time to defecation and the Bristol Stool form Scale in pre and post-treatment were 4.33±1.88, 5.96±2.29, 12.30±7.95 min and 2.10±0.95 in pyridostigmine group and 2.96±1.81, 4.06±2.22, 6.67±5.23 min and 1.41±0.84 in bisacodyl group, respectively. The significant difference was observed in both pyridostigmine and bisacodyl groups (P=0.005, P=0.002, P=0.002 and P=0.005, respectively). 60% and 32.3 of patients in pyridostigmine and bisacodyl groups recovered from self-digitations, respectively. In pyridostigmine and bisacodyl groups, 66.7% and 32.3 of them had improvement in the sense of incomplete defecation, respectively.

**Conclusion:**

Pyridostigmine and bisacodyl significantly improved the symptoms of chronic constipation similarly.

Constipation is one of the most common chronic digestive disorders in adults (1, 2). In a self-reported survey of 1028 young adults, 52%, 44%, 32% and 20% had difficulty of straining at stools, hard stool, (feeling of being unable to completely empty during bowel movement) longer intervals between bowel movements and abdominal pain, respectively. Today, the Rome III diagnostic criteria are used to define functional constipation (3-6). Chronic constipation refers to the constipation that no response is seen after 6 months of treatment with conventional methods (7). Routine treatment of constipation includes correct diet, increasing physical activity, dietary fiber, senna combinations, laxatives, milk of magnesia, lactulose and other treatment methods (8).

Approximately 5% of patients suffer from severe or chronic constipation and they do not respond to above treatment (9). Treatment with R-met Hunt-3 lubiprostone is used for these patients (9, 10). The use of peripheral opioid antagonists are also considered in the treatment of chronic constipation (11). Many of these patients used bisacodyl, but totally some of them did not respond to the abovementioned treatment. The release of acetylcholinesterase in constipated patients or in slow-transit constipated patients is less than in patients control group (12).

By increasing acetylcholine, the gastrointestinal motility and cholinesterase inhibitors are enhanced. Cholinesterase inhibitor drugs escalate acetylcholine resulting in gastrointestinal motility (13, 14). Histoimmunological studies had shown that the delay of gastrointestinal movement in patients with constipation was caused by the decrease of acetylcholine and increase of nitric oxide (15). These observations indicate that cholinesterase inhibitors can be used to treat constipation by increasing acetylcholine.

Pyridostigmine is a reversible cholinesterase inhibitor of cholinergic drugs that prevents the degradation of acetylcholine and increases its concentration in the synapses so it facilitates the transmission of impulses from the neuromuscular junction (16). Long-acting cholinesterase inhibitors such as pyridostigmine reduce the constipation in patients with Parkinson's disease and autoimmune neuropathy (17). A study on 126 patients who suffered from post-polio syndrome showed that pyridostigmine (with low dose of 60 mg and three times a day orally) caused diarrhea in 55% of patients. However, only 12% of patients who received placebo got diarrhea. Pyridostigmine caused diarrhea in patients of this study therefore, it shows that this medication can increase intestinal motility and transit. But the problem of the current study was that the pyridostigmine was not directly used to treat the constipation of patients. However, the effect of pyridostigmine on colonic transit and chronic constipation has not been systematically evaluated yet (7).

Although nowadays bisacodyl is used as main drug in the treatment of chronic constipation in most cases, patients do not respond to this treatment in many cases and bisacodyl cannot be used for long periods, too. Long-term use of bisacodyl causes disorders in bowel movement. Side effects of bisacodyl are muscle weakness, nausea, vomiting, anorexia, diarrhea, rectal irritation (18). The observations showed that there was no therapeutic response to bisacodyl even with double recommended doses. Many patients relate the symptoms such as abdominal distension, early satiety, anorexia to constipation because of chronic constipation and constipation increases the symptoms of anal fissure or hemorrhoids in some patients. 

It is expected that the symptoms and complications associated with constipation are decreased by using a useful drug. To do so, the aim of this study was to compare the effect of bisacodyl and pyridostigmine on the treatment of chronic constipation.

## Methods

This double-blind controlled clinical trial study was conducted on patients with chronic constipation and refractory to conventional therapy. Constipation in these patients was confirmed by the Rome III criteria (4). The studied patients were older than 18 and younger than 75 years. They did not recover from constipation despite the use of conventional therapies at least for six months. All patients had similar diets and normal colonoscopy in terms of colon structure. Patients with underlying diseases that affect the motility of the gastrointestinal tract such as thyroid, diabetes, congestive heart failure, with neurological diseases like Parkinson's disease, chronic renal failure, stroke, cardiac arrhythmia, with a history of abdominal surgery (appendectomy, cholecystectomy, inguinal hernia, hysterectomy), ECG abnormalities (prolonged QT, block AV), with a history of respiratory problems and patients who used the drugs which affect the gastrointestinal tract such as tricyclic antidepressants, drugs stimulating the digestive system, calcium channel blockers and all women with the chance of pregnancy in the past 48 hours with a negative pregnancy test were excluded from the present study.

The effect of pyridostigmine and bisacodyl on the treatment of chronic constipation was compared among 68 patients. Patients were randomly divided into two groups. The first and second groups were treated using 5 mg bisacodyl and 60 mg pyridostigmine every 8 hours for 4 weeks, respectively. 

Sex distribution was the same in two groups. Before and during the treatment, the gastrointestinal symptoms including Bristol score, straining defecation, the time of defecation, the number of the number of defecation in a week, sense of incomplete defecation and self-digitations were recorded every two weeks for all patients. In Bristol stool scale, types 1 and 2 indicate constipation, types 3 and 4 show normal defecation and types 5, 6 and 7 illustrate diarrhea in patients (10, 19). Straining defecation, the time of defecation (based on minute), the number of defecation in a week, sense of incomplete defecation and self-digitations (using external objects to empty stool) were determined using the VAS score (score 0 = no pain and 10= severe pain), asking questions and gathering the history of patients. 

Variables such as age, sex, height, weight, BMI, occupation and parity were recorded using a questionnaire containing demographic data. Men and women were separately and systematically divided into 2 groups. The first and second groups were treated using 5 mg bisacodyl and 60 mg pyridostigmine every 8 hours for 4 weeks, respectively. A dose of 60 mg pyridostigmine was prescribed 3 times a day since the bioavailability of pyridostigmine is 11.5%-18/9%, this drug is extracted by the kidneys and the therapeutic dose of pyridostigmine in myasthenia gravis varies between 60 to 1500 mg per day. In this study, the dose of pyridostigmine was determined 60 mg every 8 hours and a total of 180 mg daily (20) because the Tmax of this drug is 2-1 hours and its effect is 6-3 hours. To prevent any systematic error, double-blind study was done. Due to the differences in the appearance of drugs, all drugs were encapsulated in uniform capsules and then were coded so that neither patients nor physicians were aware of the type of administration.

Finally, all data were analyzed using SPSS software. Differences between variables were examined using t-test using test and paired t-test and difference in each case was considered significant with p-value less than 0.05.

## Results

Sixty-eight patents with mean age of 49.84±12.6 years (58.8% females) entered to study the characteristics of the patient and control groups are presented in table 1. Both pyridostigmine and bisacodyl groups were the same in terms of age, weight, height, BMI, number of smokers, parity, duration of constipation and education and there was no significant difference between two groups (table 1).

**Table 1:** Baseline characteristics

**Variable** **Treatment group**		**Bisacodyl**	**Pyridostigmine**
gender	Male (No)	14	14
	Female (No)	20	20
Age(year)		50.47±12.09	13.38±49.21
Height(cm)		8.3±163.7	7.3±165.2
Weight(kg) (Mean±SD)		11.58±70.91	11.64±70.91
BMI(kg/m^2^)		26.53	25.92
Smoker(n)		4	5
Delivery(n)	NVD	14	14
	cesarean	5	7
Duration of constipation(year)		10.06±11.61	7.49±10.23
Education	illiterate	10	6
	High school	11	12
	diploma	6	12
	graduated	7	4

The mean number of defecation per week was 1.55±1.28 and 2.26±1.48 in pyridostigmine and bisacodyl groups, respectively. In addition, it reached to 5.96±1.84 and 5.16±1.95 after 4 weeks of treatment in the pyridostigmine bisacodyl groups, respectively. The mean difference of defecation frequency per week was 4.33±1.88 and 2.96±1.81 in pyridostigmine and bisacodyl groups before and after treatment, respectively. There was significant difference in pyridostigmine group than bisacodyl group (P=0.005). The mean VAS score at baseline for straining was 9.58±0.95 in pyridostigmine group and 8.70±1.76 in bisacodyl group and after 4 weeks of treatment, it was 3.6±2.26 and 4.70±2.03 in the pyridostigmine and the bisacodyl groups, respectively.

Before and after treatment, the mean difference of VAS score was 5.96±2.29 and 4.06±2.22 in pyridostigmine and bisacodyl groups, respectively, that a significant difference was observed in pyridostigmine group (P=0.002). The mean of the time defecation was 17.91±8.19 and 14.97±10.85 min in pyridostigmine and bisacodyl groups, respectively before the study. After 4 weeks of treatment, it was 5.00±4.46 minutes in pyridostigmine group and 8.93±11.40 minutes in bisacodyl group. The mean difference of time defecation before and after treatment was 12.30±7.95 min in pyridostigmine group and 6.67±5.23 min in bisacodyl, which indicated a significant difference in pyridostigmine group compared to bisacodyl (P=0.002).

The mean of Bristol stool scale in patients was 1.29±0.52 and 1.73±0.75 before the treatment in pyridostigmine and bisacodyl groups, respectively, and after 4 weeks of treatment with the pyridostigmine and bisacodyl drugs, it reached to 3.43±1.00) and 3.16±0.86), respectively. The mean difference of Bristol stool scale was 2.10±0.95 and 1.41±0.84 in pyridostigmine and bisacodyl groups during pre and post-treatment, respectively. There was a significant difference in the group of pyridostigmine to bisacodyl group (P=0.005) (table 2).

**Table 2.** Frequency, mean and standard deviation of the difference between two groups of patients with refractory constipation taking bisacodyl and pyridostigmine before and after treatment

**variable**	**drug**	**n**	**mean**	**Standard deviation**	**P value**
Bristol Score difference	Pyridostigmine	30	2.10	.95	0.005
	Bisacodyl	31	1.41	.84	
defecation in week difference(n)	Pyridostigmine	30	4.33	1.88	0.005
	Bisacodyl	31	2.96	1.81	
VAS score difference	Pyridostigmine	30	5.96	2.29	0.002
	Bisacodyl	31	4.06	2.22	
Time of defecation difference	Pyridostigmine	30	12.30	7.95	0.002
	Bisacodyl	31	6.67	5.23	

**Fig 1:**
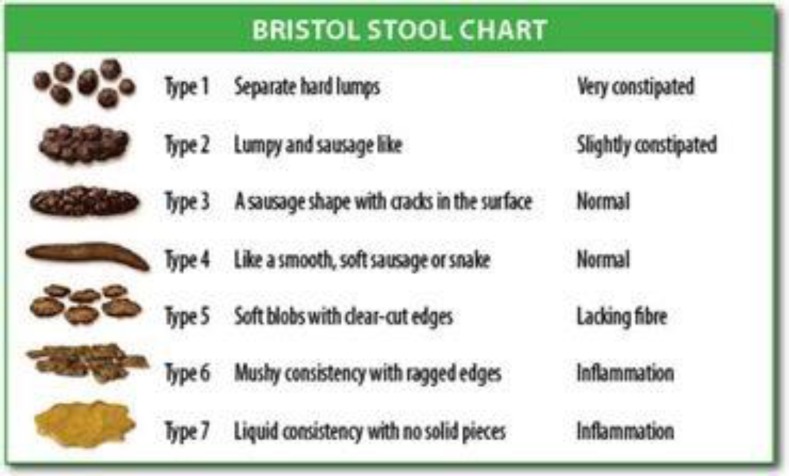
Bristol stool form scale

At the beginning of the study, 25 and 18 patients in pyridostigmine and bisacodyl groups had self-digitation, respectively. After 4 weeks of treatment with bisacodyl and pyridostigmine, 6 and 3 patients had self-digitation, respectively. 60% of patients in pyridostigmine group and 32.3 in bisacodyl group recovered from self-digitations (figure 1). At baseline of the study, 34 patients of pyridostigmine and 33 patients of bisacodyl groups had a sense of the incomplete defecation. After 4 weeks, 10 and 20 patients of pyridostigmine and bisacodyl groups had a sense of incomplete defecation. 66.7% and 32.3% patients of pyridostigmine and bisacodyl groups had improvement in the sense of incomplete defecation, respectively (figure 1).

There was a significant difference among the patients with chronic constipation in pyridostigmine and bisacodyl groups (P=0.001). 

It is obvious that the therapeutic differences were higher in the pyridostigmine group than the bisacodyl group.

The questionnaires of four patients in pyridostigmine group and three patients in bisacodyl group were not completed in the third session. The therapeutic dose of a patient in each group was reduced to once daily.

## Discussion

The results of this study demonstrated that pyridostigmine significantly improved time to defection, and the number of defecation gastrointestinal symptoms compared with bisacodyl. Pyridostigmine (a cholinesterase inhibitor) was well tolerated and improved the symptoms in patients. VAS score, self-digitation, the time of defecation, sense of incomplete defecation and the number of defecation in a week significantly improved the pyridostigmine group compared to the bisacodyl group. Although the changes in bisacodyl group showed the improvement, these changes in pyridostigmine group were significantly higher than the bisacodyl group.

Results of a study in 2013 indicated that pyridostigmine improves gastrointestinal symptoms and accelerates the movement of the colon in patients with diabetes and constipation (21). These results are similar to those of the present study. Pyridostigmine increased colon movement speed by 0.5 units in other studies in patients with chronic constipation and IBS. This increase was considerable compared with linaclotide (0.4-unit increase), serotonin 5-HT4 agonists such tegaserod (0.4-unit increase) and r (0.6-unit increase) (22, 23).

In the current study, pyridostigmine produced averagely 2-unit change in patients with Bristol stool form scale that this change was 0.9 and less than 0.9 of units with renzapride and linaclotide, respectively (20, 24). In a study, acute intravenous neostigmine and oral pyridostigmine were effective on the move of colonies in patients with autoimmune neuropathy and constipation. This study suggested that cholinesterase inhibitors could accelerate the motion of colon (25). A study in 2008 showed that pyridostigmine could improve colon movements and symptoms in patients with autoimmune neuropathy and constipation (7). The same result was observed in the present study. 

In one similar study indicated that the use of linaclotide (a cholinesterase inhibitor) enhanced colonic activity in the antrum of the stomach of dogs (26). In a study conducted in 2008, the use of pyridostigmine did not create any change in the symptoms of patients with slow transit constipation while it improved the symptoms in patients with recurrent pseudo-obstruction (27). A conducted study in 2012 explained that pyridostigmine improved the symptoms of bloating, early satiety and nausea in patients with dyspepsia (28).

 A dose of 180 mg pyridostigmine in this study was well tolerated by patients except one who had nausea and vomiting in the second week of the study and did not continue this process. Also, pyridostigmine dose was declined to once daily for a patient in the second week. Cholinergic and cardiovascular side effects were not observed in any patient. Other studies showed that the use of cholinesterase inhibitors could cause Gulf War Veterans’ disease (29), but this disease was not observed in the present study. Although the relationship between plasma concentration of pyridostigmine and its effect on neuromuscular function is linear, the correlation between plasma levels of pyridostigmine and its effects on the digestive system has not been studied (30).

In summary, this study suggests that the use of pyridostigmine increases the movement speed, improves the gastrointestinal symptoms and the chronic constipation in patients.
